# Biomechanical comparison of different internal fixation devices for transversely unstable Mason type II radial head fractures

**DOI:** 10.3389/fbioe.2023.1259496

**Published:** 2023-12-18

**Authors:** Xiang Zhang, Ling Gong, Hong Ma, Jinhui Liu, Xin Duan

**Affiliations:** ^1^ Department of Orthopedic Surgery, West China Hospital, Sichuan University, Chengdu, China; ^2^ Department of Health Management Center, General Practice Center, West China Hospital, Sichuan University, Chengdu, China; ^3^ Sichuan Provincial Laboratory of Orthopaedic Engineering, Department of Bone and Joint Surgery, Affiliated Hospital of Southwest Medical University, Luzhou, China; ^4^ Department of Orthopedic Surgery, Sichuan Fifth People’s Hospital, Chengdu, China

**Keywords:** transversely unstable radial head fractures, headless compression cannulated screw, tripod technique, mini-T plate, biomechanics

## Abstract

**Background:** The integrity of the radial head is critical to maintaining elbow joint stability. For radial head fractures requiring surgical treatment, headless compression cannulated screw fixation is a less invasive scheme that has fewer complications. The aim of this study was to compare the mechanical stability of different fixation devices, including headless compression cannulated screws and mini-T-plates, for the fixation of transversely unstable radial head fractures.

**Methods:** Forty identical synthetic radius bones were used to construct transverse unstable radial head fracture models. Parallel, cross, and tripod headless compression cannulated screw fixation and mini-T plate fixation were applied. The structural stiffness of each group was compared by static shear loading. Afterward, cyclic loading was performed in each of the three directions of the radial head, and the shear stability of each group was compared by calculating the maximum radial head displacement at the end of the cycle.

**Findings:** The mini-T plate group had the lowest structural stiffness (51.8 ± 7.7 N/mm) and the highest relative displacement of the radial head after cyclic loading (*p* < 0.05). The tripod headless compression cannulated screw group had the highest structural stiffness among all screw groups (*p* < 0.05). However, there was no significant difference in the relative displacement of the radial head between the screw groups after cyclic loading in different directions (*p* > 0.05).

**Interpretation:** In conclusion, the biomechanical stability of the mini-T plate for fixation of transverse unstable radial head fractures is lower than that of headless compression cannulated screws. Tripod fixation provides more stable fixation than parallel and cross fixation with headless compression cannulated screws for the treatment of transversely unstable radial head fractures.

## 1 Introduction

Radial head fracture is a common elbow injury, accounting for approximately 30% of elbow joint fractures (Lacheta et al., 2019; Sun et al., 2022). It is caused by the radial head slamming against the condyles of the humerus when the upper limb is propped up on the ground in a rotating forward external booth (Meacher et al., 2020). The radial head is an important component of the elbow joint, and its integrity directly affects the stability of the elbow joint and forearm function (Kodde et al., 2020). Therefore, it is critical to effectively restore the anatomic structure and ensure joint stability of the radial head and surrounding tissues after determining the specific stage.

In 1954, Mason (Mason, 1954) first proposed the classification of radial head fractures, which was modified by Broberg and Morrey (Broberg and Morrey, 1987), resulting in the most common current classification of “Mason fractures.” However, there is no consensus on the treatment of transversely unstable Mason II radial head fractures (M2RFs), but open reduction and internal fixation (ORIF), including screws, plates, and kerf pins, is the trend. Currently, most recommendations are based on expert opinion and lack support from scientific evidence. The incidence of complications such as internal fixation failure (Xu et al., 2020) and fracture nonunion (Golinvaux et al., 2020) remain high, and the initial stability of the fracture break is one of the main influencing factors. In the past, such fractures were fixed with plates, especially mini-T plates (MTPs), because of their small size, thin plate surface, and simple application (Yang et al., 2023). Moreover, plates have been shown to lead to favorable biomechanical outcomes in the treatment of axially unstable radial head fractures (Yang et al., 2023). Recently, headless compression cannulated screws (HCCSs) have been used for the treatment of M2RFs because of their advantages such as greater fixation strength and unrestricted fixation position (Al-Tawil and Arya, 2021; Gray et al., 2022). However, it is uncertain whether MTP or HCCSs are biomechanically superior in the treatment of transversely unstable M2RFs.

The aim of this study was to compare the strength of the fixation of transversely unstable M2RFs by using biomechanical testing (BT) with parallel cross-implantation of two HCCSs, tripod technique of three HCCSs, and MTPs to provide a biomechanical basis for the selection of internal fixation devices for the surgical treatment of transversely unstable M2RFs.

## 2 Methods

### 2.1 Specimens

**FIGURE 1:**
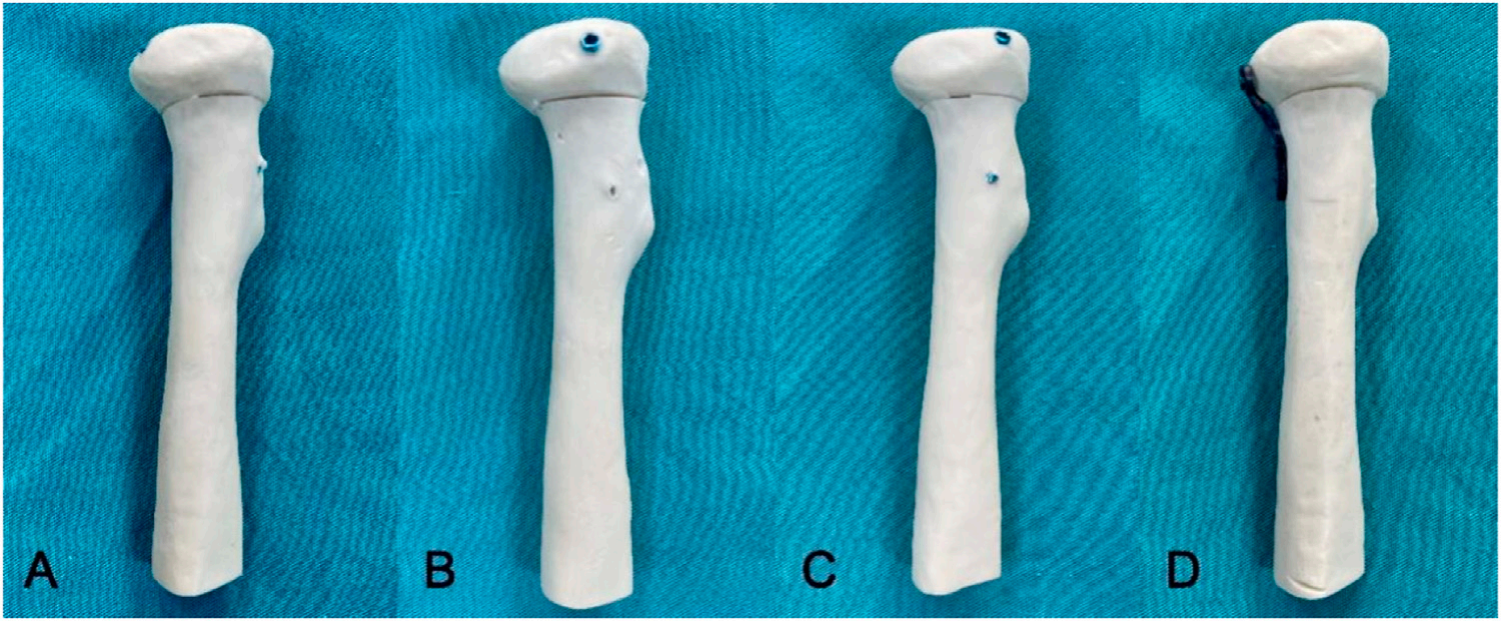
The transversely unstable M2RF models in each group. **(A)** G-PS group: two HCCSs were inserted parallel to the top outer edge of the radial head at 45° to the radial head axis; **(B)** G-CS group: two HCCSs were placed approximately 60° apart and buried below the articular cartilage surface; **(C)**: G-TS group: three HCCSs were distributed circumferentially around the radial head to form a tripod; **(D)**: G-0TP group: the MTP was prebent and shaped according to the radial head and placed in the radial safety zone.

Forty synthetic radius bones of identical size and density (Synbone 7220, Synbone AG) were used in this study. These synthetic radius bones were 255 mm in length, with a 13° valgus angle and a radial head diameter of 27 mm. In this study, transversely unstable radial head fractures were established according to the experimental protocol of Chen et al. (Chen et al., 2017). A miniature table saw was used to make osteotomies at the junction of the radial head and radial neck in each specimen to ensure consistent fracture modeling in each group. Moreover, we intercepted the proximal 10 cm of the radius for biomechanical experiments and fixed each group of M2RF models using HCCSs (Arthrex Corporation, United States) and a MTP (Synthes Corporation, United States). These specimens were divided into the following four groups (Figure 1) according to the internal fixation method: parallel HCCSs group (G-PS), crossed HCCSs group (G-CS), tripod HCCSs group (G-TS) and MTP group (G-TP).

### 2.2 Surgical technique

**FIGURE 2:**
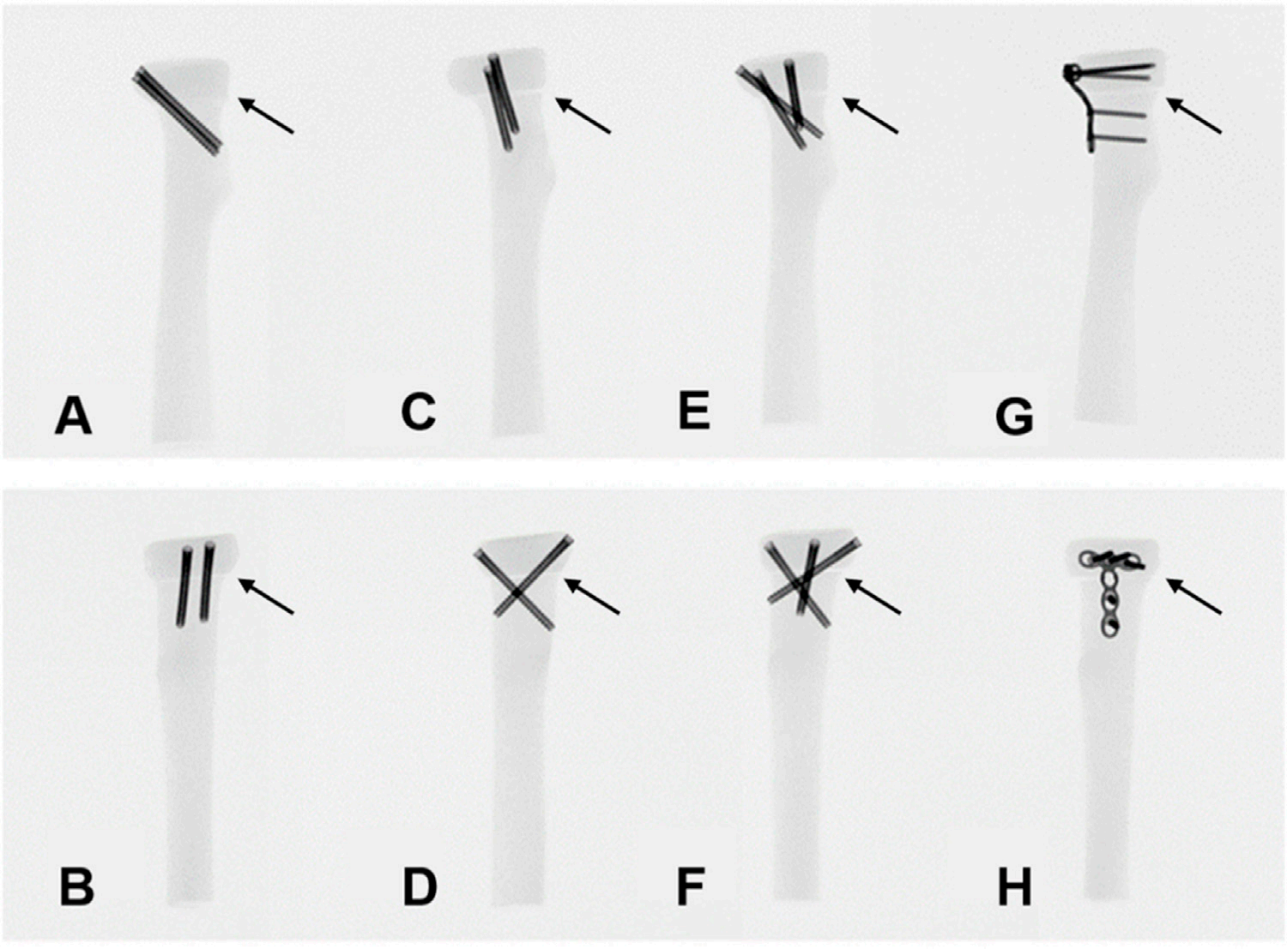
Anteroposterior and lateral views of the X-ray films in each group. **(A,B)** G-PS group; **(C,D)** G-CS group; **(E,F)** G-TS group; **(G,H)** G-TP group. Arrows indicate the position of the fracture line in the fracture models.

Osteosynthesis was performed for all the specimens by the same surgeon. In G-PS, two 3.0 mm HCCSs were inserted parallel to the top outer edge of the radial head at 45° to the radial head axis, with the two screws being the same length and approximately 5 mm apart. In G-CS, the two HCCSs were placed approximately 60° apart and buried below the articular cartilage surface. The screw tip was also placed less than 2 mm beyond the contralateral cortex to avoid interference with the ulna. In G-TS, based on the tripod technique proposed by Lipman et al., 2018, three HCCSs were distributed circumferentially around the radial head to form a tripod. In G-TP, the 2.0 mm MTP was prebent and shaped according to the anatomy of the radial head and placed in the radial safety zone, located dorsal to the radius. The position of the internal fixation was evaluated using X-ray. Radiological examination of transversely unstable M2RF models fixed by the four fixation methods mentioned above was performed using a C-arm X-ray machine (Ziehm Imaging, Germany) (Figure 2).

### 2.3 Static shear experiment

**FIGURE 3:**
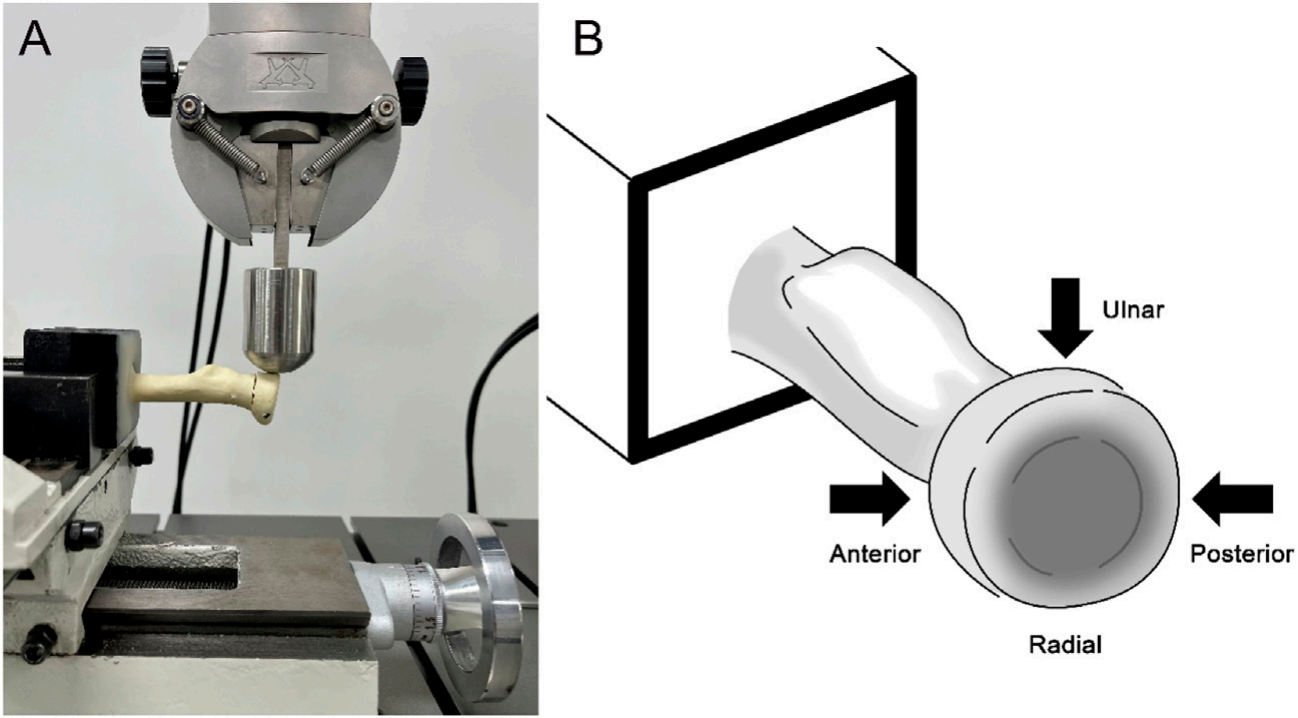
Experimental test setup. **(A)** The transversely unstable M2RF models were fixed to the experimental table to apply shear loads; **(B)** Shear loads were applied to each sample in each of the three directions to simulate the shear force exerted by the ulna on the radial head in the sigmoid notch during forearm rotation.

A total of 20 transversely unstable M2RF models were randomly selected from each group for the static shear experiment. Testing was conducted by the INSTRON universal mechanical testing machine (INSTRON Corporation, United States) (Figure 3). The distal ends of the fracture models were fixed to the experimental table using custom-made clamps. A shearing force was applied to the radial head fragment at a distance of 5 mm from the fracture line at a rate of 2 mm/min from the posterior to the anterior side of the radius (Chen et al., 2017). In this way, the shear force from the ulnar sigmoid notch was simulated during the rotation of the radial head. The experiment was stopped when the radial head became displaced by 2 mm or the internal fixation failed, and the force‒displacement curves in each group were plotted to analyze the structural stiffness and overall stability. Then, the structural stiffness of each model was calculated by the force‒displacement curves. Implant failure was defined as (Wang et al., 2018) 1) the appearance of a new fracture line in the model in addition to the original fracture line; 2) bending, cutting out, or fracture of MTPs or HCCSs; 3) shear displacement of the radial head exceeding 2 mm; and 4) flattening of the force‒displacement curve in the data acquisition system or no significant change in the displacement of the model when the load continued to be increased.

### 2.4 Cycle shear experiment

The remaining 4 groups of 20 transversely unstable M2RF models were used for the low-cycle shear experiment. A cyclic loading scheme of 4 Hz was set in the software Instron Wave Matrix2 (INSTRON Corporation, United States), and 300 shear loading cycles were applied to obtain information about the relative displacement of the fragments (Wagner et al., 2020). A load of 20 N was applied sequentially in each of the three loading directions: posterior to anterior (P-A), ulnar to radial (U-R), and anterior to posterior (A-P), to simulate the shear load generated by the ulnar sigmoid tuberosity on the radial head during anterior-posterior rotation of the forearm. The relative displacement of the radial head at the end of the cyclic shear test at each load application point and the maximum displacement in each cyclic cycle were recorded. The time‒displacement curves of each group were analyzed, and the shear stability of each group was compared by the maximum displacement of the last cycle loading.

### 2.5 Statistical analysis

Statistical analysis was conducted using GraphPad Prism 9. First, the Shapiro‒Wilk test was used to test whether the data of each group coincided with a normal distribution, and if the data coincided with a normal distribution, the *t*-test was used to compare the data between groups; if not, the rank sum test was used to analyze the data between groups. *p* < 0.05 was defined as statistically significant.

## 3 Results

### 3.1 Construct stiffness

**FIGURE 4:**
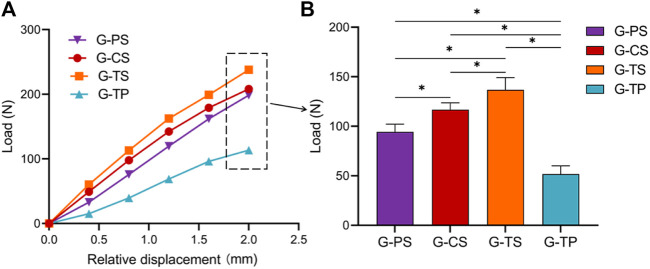
Comparison of shear stiffness of a total of 40 experimental samples from the G-PS, G-CS, G-TS and G-TP groups. **(A)** Four groups of displacement‒load curves, where the slope of the curve represents the shear stiffness of each group; **(B)** Load value of each group when the displacement reaches 2 mm. The standard deviation is represented with the range bars on top of each graph. (*p < 0.05).

The results of the static shear experiment indicated that the G-TP showed the weakest shear stiffness among the groups with 51.8 ± 7.7 N/mm, while the G-TS exhibited the strongest shear stiffness with 136.8 ± 11.8 N/mm. Moreover, the mean value of shear stiffness was 94.4 ± 7.4 N/mm in the G-PS and 116.7 ± 6.7 N/mm in the G-CS. There was a statistically significant difference in shear stiffness among the four groups (Figure 4).

### 3.2 Changes after cyclic loading

**FIGURE 5:**
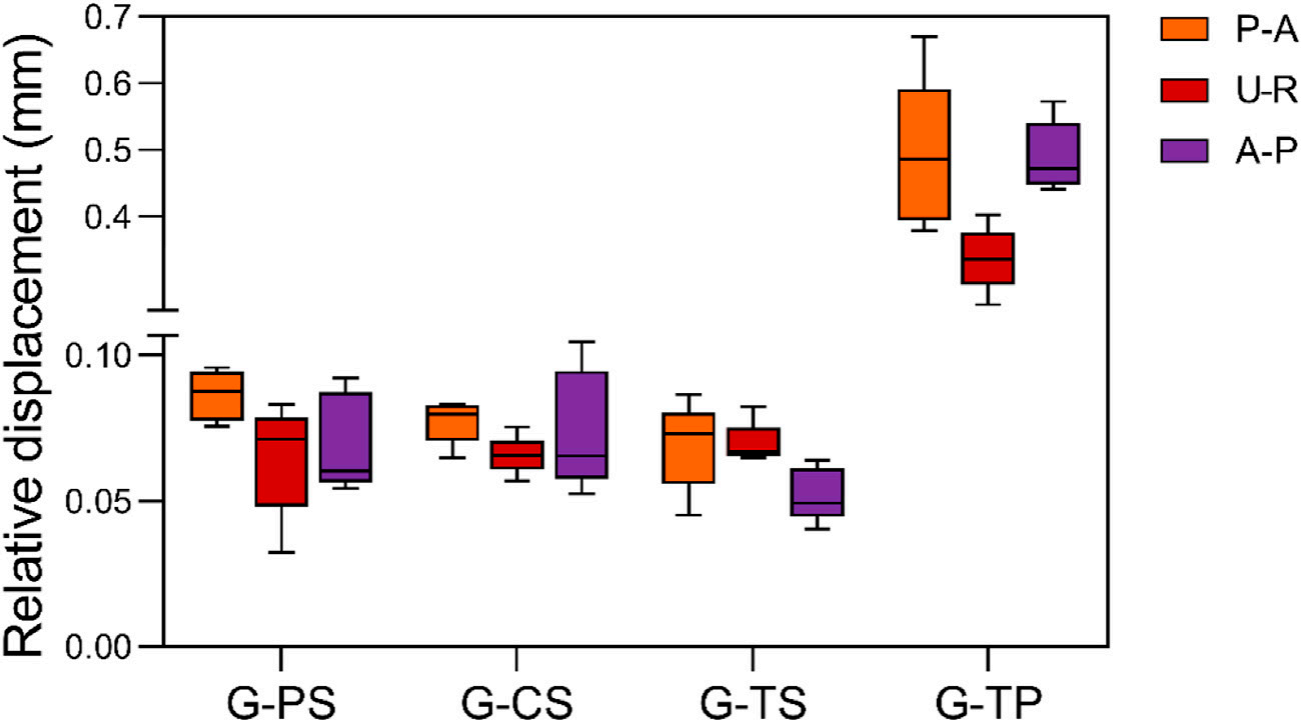
Maximum relative displacement of each group in each of the three loading directions of posterior to anterior (P-A), ulnar to radial (U-R), and anterior to posterior (A-P) after cyclic load loading.

**FIGURE 6:**
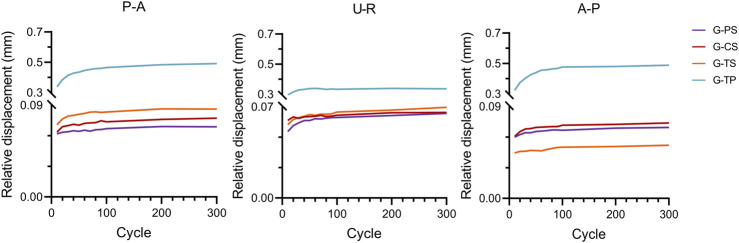
Peak relative displacement curve for the G-PS, G-CS, G-TS, and G-TP groups in each of the three loading directions of posterior to anterior (P-A), ulnar to radial (U-R), and anterior to posterior (A-P) during the cyclic shear experiment.

The relative displacement of the radial head in the G-PS was 0.086 ± 0.008 mm (P-A), 0.065 ± 0.017 mm (U-R), and 0.070 ± 0.015 mm (A-P); in the G-CS was 0.077 ± 0.007 mm (P-A), 0.066 ± 0.015 mm (U-R) and 0.074 ± 0.019 mm (A-P), respectively; in the G-TS was 0.069 ± 0.014 mm (P-A), 0.070 ± 0.006 mm (U-R) and 0.052 ± 0.008 mm (A-P), respectively; in the G-TP was 0.492 ± 0.102 mm (P-A), 0.337 ± 0.043 mm (U-R) and 0.489 ± 0.047 (A-P), respectively (Table 1). After the low cycle loading test, in the P-A direction, the G-TS showed the smallest relative displacement among all groups, and the difference was statistically significant; in the U-R direction, the G-PS exhibited the smallest relative displacement; in the A-P direction, the G-TS showed the smallest relative displacement. Moreover, each cycle during low cycle loading at different positions indicated that the maximum displacement of the G-TP was greater than that of all HCCs groups (Figures 5, 6).

**TABLE 1:** Relative displacement of the radial head in each of the three loading directions of posterior to anterior (P-A), ulnar to radial (U-R), and anterior to posterior (A-P) at the end of the cyclic shear test in the G-PS, G-CS, G-TS and G-TP groups.

	G-PS	G-CS	G-TS	G-TP
P-A (mm)	0.086 ± 0.008	0.077 ± 0.007	0.069 ± 0.014	0.492 ± 0.102
U-R (mm)	0.065 ± 0.017	0.066 ± 0.015	0.070 ± 0.006	0.337 ± 0.043
A-P (mm)	0.070 ± 0.015	0.074 ± 0.019	0.052 ± 0.008	0.489 ± 0.047

## 4 Discussion

Conservative treatment for M2RFs predisposes patients to complications such as elbow pain, decreased grip strength, and lateral elbow instability because of the inability to achieve fracture end reduction (Nietschke et al., 2018; Wen et al., 2022). It has long been accepted that M2RF with displacement ≥2 mm requires ORIF (Ruchelsman et al., 2013). Zarattini et al. (Zarattini et al., 2012) conducted a 10-year retrospective controlled study of M2RFs and reported less residual pain and greater joint mobility in M2RF patients who underwent ORIF. Maintaining the stability of radial head fractures remains a challenge for orthopedic surgeons due to the potential to cause problems such as combined elbow injuries (Mellema et al., 2016). Studies have shown that a MTP and HCCs have favorable biomechanical outcomes in fixing axially unstable radial head fractures (Demiroglu et al., 2016; Yang et al., 2023; Zhang et al., 2023). However, it is uncertain whether MTP or HCCSs have a biomechanical advantage in fixing transversely unstable radial head fractures. Long-term stable fixation not only promotes early postoperative functional exercise and accelerated elbow rehabilitation but also reduces the incidence of complications. Therefore, in this study, we investigated the biomechanical stability of four different internal fixation methods for the treatment of transversely unstable M2RFs, which provided a biomechanical basis for the choice of internal fixation options for the treatment of M2RFs.

In the static shear experiment, the structural stiffness of each HCCSs group was significantly greater than that of the MTP group, indicating that the HCCSs provided better shear resistance than the MTP. This may be because the HCCSs in each group crossed the fracture end on both sides and gave continuous compression to the fracture surface, and the MTP may lack a rigid connection at the fracture. Moreover, Zhang et al., 2023 conducted a study of follow-up outcomes after ORIF of M2RFs and found that fixation with MTP was associated with higher incidences (10-fold) of complications and reoperations than screws alone. The position and protrusion of the plate are among the important contributing factors. The greatest advantage of HCCSs is that they can be inserted into any part of the radial head with minimal dissection and provide considerable biomechanical stability. Hence, fixation with HCCSs is preferred, especially for transversely unstable M2RFs. Compared to G-PS and G-CS, G-TS showed the greatest stiffness and less relative displacement under low cyclic shear loading, showing the better biomechanical stability of three HCCSs for fixation of transversely unstable M2RFs with tripod-type implantation. Model et al., 2022 followed 13 patients with radial neck fractures treated with HCCSs for up to 72 months and showed that the tripod technique is an effective alternative to conventional plate screw fixation of unstable M2RFs. A biomechanical study by Rebgetz et al., 2019 showed that there was no difference in fixation stiffness between the HCCSs placed in a tripod-type design and the locking plate for axially unstable M3RFs, and one of the main drawback associated with plate fixation was soft tissue irritation. This experiment expands current knowledge regarding biomechanical experiments of transversely unstable M2RFs, providing valid biomechanical evidence. In addition, although G-CS exhibited less stiffness and relative displacement than G-TS, it is also commonly used in clinical practice to treat fractures of the radial head and radial neck. Gutowski et al. (2015) used a simple biomechanical experiment to compare the mechanical performance of crossed screw and plate fixation for radial neck fractures and showed that both internal fixation modalities exhibited similar biomechanical stability in the treatment of transverse, noncomminuted radial neck fractures.

Interestingly, the relative displacements of each HCCS group in the three directions were not significantly different. Therefore, it can be speculated that to some extent, when increasing the number of screws or changing the screw fixation method, the relative displacement and stiffness of the fracture end do not change significantly. Tarallo et al., 2018 reviewed the clinical and radiographic data of 61 patients with radial head fractures treated with mini-screws and found that varying numbers of mini-screws provided adequate strength and stiffness in patients with radial head fractures and that these patients had good clinical and functional scores at the mid-term follow-up. This suggests that the number of screws and the method of fixation may have little effect on the overall stability of the elbow joint. Amanatullah et al., 2012 evaluated the biomechanical performance of three screw orientations for the fixation of vertical shear fractures of the inner ankle. They concluded that when the screws are not placed parallel, only the first screw produces a compression effect, and any nonparallel screws do not add additional compression but serve to stabilize rotation, translation, etc. Similar conclusions can be drawn from our results: when the first screw acts as a compression, the additional implanted HCCSs act as a resistance to fracture surface translation in the P-A and U-R directions. However, parallel fixation of the fracture ends seems to provide sufficient resistance to shear forces, and increasing the number of screws or changing the screw configuration to increase the resistance to rotation and translation is not significant enough, which requires further confirmation by experiments with applied torsional loads. Since the biomechanical stability of the G-CS and G-TS was not significantly improved compared to that of the G-PS, operators may consider the use of two HCCSs implanted in parallel to fix the fracture ends when treating transversely unstable M2RFs. During surgical treatment, implantation of HCCSs, either in a crossed or tripod fashion, does not allow simultaneous exposure of the implantation site of the HCCSs if the forearm cannot be rotated (Model et al., 2022; Yano et al., 2023). Especially with tripod implantation of HCCSs, interference between screws may occur. Moreover, the stability of the internal fixation may be reduced during repeated retraction of the screws to adjust their optimal position. In contrast, parallel implantation of HCCSs requires only a small incision and fixation of the fracture break to the radial shaft at a 45° angle, without rotation of the forearm. Therefore, in the surgical treatment of transversely unstable radial head fractures, parallel screw fixation may have the advantage of simpler manipulation and an even shorter operative time while providing the same fixation strength.

Most of the current biomechanical experiments on M2RFs are static experiments, and there are almost no cycle loading experiments to simulate postoperative functional exercise. Additionally, this study simulated the mechanics of postoperative functional exercise for transversely unstable M2RFs through low circulation shear experiments, providing a biomechanical basis for the selection of internal fixation protocols for clinical treatment. From the results of the cycle loading experiments, the maximum relative displacement values of the HCCS groups were significantly smaller than those of the MTP group (Figure 6). This indicated that during postoperative forearm rotation, the stability of HCCSs for transversely unstable M2RFs was significantly better than that of MTP under the cyclic action of shear resistance. The MTP fixated only the radial side of the fracture end and lacked the compression effect on the fracture end, similar to the HCCSs; therefore, the relative displacement was significantly greater when subjected to shear forces. In addition, it is noteworthy that the relative displacements of each HCCS in different directions did not differ significantly after experiencing low cycle shear loading in different directions, and the difference was not statistically significant. Giffin et al., 2004 performed *in vitro* biomechanical experiments on crossed screws as well as plates for radial neck fractures, and the results showed that different orientations did not significantly affect the stiffness of various internal fixations, which is consistent with our findings. The relative displacements of the HCCSs group and the maximum displacements of each loading cycle under shear loads in different directions were significantly smaller than those of the MTP group, and no internal fixation failure occurred, which further proved the reliability of HCCSs for transversely unstable M2RFs.

Axial compression testing does not demonstrate variability between internal fixation techniques because the fracture ends compress against each other when axial loading is applied, and little mechanical difference is produced between groups. In addition, torsional loading is unlikely to be the cause of internal fixation failure due to the lack of restraint at the proximal end of the radius. Therefore, we chose multidirectional shear loading and conducted experiments by low-cycle loading and static tests to reach the conclusions of this study. Increasing the sample size may help to identify subtle differences between different internal fixation methods. In addition, the present study has some limitations. Soft tissues such as muscles and ligaments were not simulated in this experiment to mimic the mechanical characteristics of the real human elbow joint. They also play an important role in the overall stability of the elbow joint (Strafun et al., 2018). However, most of the biomechanical experiments simplified the design of the experimental protocols to perform biomechanical experiments on radial head fractures *in vitro* (Burkhart et al., 2007; Burkhart et al., 2010; Gutowski et al., 2015), which supports the comparability of this study.

## 5 Conclusion

HCCS fixation for the treatment of laterally unstable M2RFs is a simple, minimally invasive surgical procedure that demonstrates better stability than mini-T-plates during simulated postoperative forearm rotation. Among the four internal fixation strategies analyzed in this study, tripod HCCS fixation was the best choice for the treatment of laterally unstable M2RFs. Specifically, in the surgical treatment of transversely unstable radial head fractures, we recommend tripod HCCS fixation. In addition, the conclusions of this study need to be validated by a large number of clinical studies.

## Data Availability

The raw data supporting the conclusion of this article will be made available by the authors, without undue reservation.
